# Onychomycosis in KwaZulu-Natal: Clinical spectrum, risk factors and fungal susceptibility

**DOI:** 10.4102/jcmsa.v4i1.409

**Published:** 2026-07-20

**Authors:** Jaya Jhoomuck, Yesholata Mahabeer, Antoinette Chateau

**Affiliations:** 1 Discipline of Dermatology, Nelson R Mandela School of Clinical Medicine, University of KwaZulu-Natal, Durban, South Africa; 2 Department of Medical Microbiology, National Health Laboratory Service, Durban, South Africa; 3 Discipline of Medical Microbiology, School of Medicine, College of Health Sciences, University of KwaZulu-Natal, Durban, South Africa; 4 Department of Dermatology, Grey’s Hospital, Pietermaritzburg, South Africa

**Keywords:** onychomycosis, fluconazole, demographics, risk factors, causative species, KwaZulu-Natal, South Africa

## Abstract

**Background:**

Although onychomycosis is common worldwide and often significantly impairs the quality of life of patients, local epidemiological and mycological data are lacking. We therefore investigated the risk factors, clinical features and mycological aspects of onychomycosis in KwaZulu-Natal.

**Methods:**

This study had two components. In the retrospective arm, positive fungal nail cultures from 2018 to 2024 were analysed. Prospectively in 2025, clinical and mycological data were collated. Descriptive statistical methods were used to summarise the dataset.

**Results:**

Out of a total of 301 patients, 229 patients with positive cultures were analysed. The retrospective analysis (*n* = 177) showed *Candida* species were the most common (66.7%), followed by moulds (13.6%) and *Trichophyton* species (11.9%). Prospectively, 124 patients were included; 51.6% females and 48.4% males with a median age of 58.0 years. *Candida* species (40.4%), moulds and *Trichophyton* species (25.0% each) were most frequent in 52 patients with positive cultures. All *Candida* species were susceptible to fluconazole. Most patients had subungual hyperkeratinisation. Immunosuppression (other than human immunodeficiency virus [HIV] infection and diabetes mellitus) was the most common risk factor.

**Conclusion:**

*Candida* species were the commonest cause of onychomycosis; therefore fluconazole is an appropriate treatment modality. However, revision of the existing essential drug list is recommended to include itraconazole or terbinafine for patients with confirmed infection with moulds or dermatophytes.

**Contribution:**

This is the first detailed analysis of onychomycosis in KwaZulu-Natal and contributes to the limited body of data in South Africa. Our results may be used to inform therapeutic recommendations.

## Introduction

Onychomycosis, classified as a dermatomycosis, is a fungal infection of the nail resulting in the discolouration, thickening, disintegration and hardening of the nail bed.^1,2^ The prevalence of onychomycosis is 5.5% of the world population, representing 20% – 40% of all onychopathies and approximately 30% of cutaneous mycotic infections.^3^ The condition can be painful, interfere with daily activities, result in impaired or lost tactile functions and significantly impair the quality of life, negatively affect self-esteem, leading to social embarrassment and potentially resulting in serious complications.^2,4,5^

Multiple predisposing factors are reported to be associated with the development of onychomycosis.^6^ Dermatological risk factors include tinea pedis, psoriasis and hyperhidrosis, as well as other comorbidities such as diabetes, immunosuppression, chronic venous insufficiency, peripheral artery disease and obesity.^6^ The prevalence of onychomycosis is 25% higher in human immunodeficiency virus (HIV) infected people.^1^ Other risk factors are trauma to the nail, wearing occlusive footwear, advanced age, contact with household members who have onychomycosis or tinea pedis, hallux valgus, smoking, humidity and genetic predisposition.^6,7^

There are six clinical types of onychomycosis documented in the literature.^5,8^ Distal and lateral subungual onychomycosis is the most common type, with the infection starting in the hyponychium and distal or lateral nail bed and travelling proximally.^9^ Proximal subungual onychomycosis (PSO), commonly seen in immunocompromised patients, occurs when organisms invade the proximal nail fold and cuticle area, penetrate the new nail and migrate distally.^10^ White superficial onychomycosis occurs when pathogens invade the top layer of the nail plate directly and is clinically recognised by white ‘islands’ that spread as the disease progresses.^10^ *Candida* nail infections may first appear as oedematous, erythematous paronychia, penetrating the nail plate only after it has attacked the soft tissue around the nail.^10^ Endonychial onychomycosis, which is a fungal infection of the nail plate, occurs very rarely.^8^ The nail is discoloured, white, without recognisable subungual hyperkeratosis and onycholysis.^8^ If the entire nail is infected and pushed up by subungual hyperkeratosis, resulting in onycholysis, it is called total dystrophic onychomycosis.^8^

Onychomycosis is caused by pathogenic dermatophytes, which include *Trichophyton rubrum, Trichophyton mentagrophytes*, and *Epidermophyton floccossum*. It can also be caused by yeasts, namely *Candida albicans, Candida parapsilosis* and *Candida guilliermondii*, and by non-dermatophyte moulds (NDMs), such as *Acremonium* species, *Alternaria* species, *Aspergillus* species, *Cladosporium* species, *Fusarium* species and *Scopulariopsis brevicaulis*.^7^

Superficial mycoses are becoming increasingly resistant to current antifungal medications worldwide.^11^ Current treatment regimens with systemic or topical antifungal agents do not yield satisfactory results, with more than half of the patients deemed not cured.^12^ The literature suggests that fluconazole is less effective than other oral antifungals, such as itraconazole, ketoconazole, griseofulvin and terbinafine and is only prescribed when other antifungals are not tolerable.^1^ In our resource-limited setting, fluconazole is the only available oral antifungal. Unfortunately, we have observed that many patients are recalcitrant to this. It is therefore imperative to determine the spectrum of fungal infections seen, the causative organisms and susceptibility to assist with targeted therapy.

Although onychomycosis is common worldwide and constitutes a significant public health concern,^2,13^ published data from South Africa remain scarce. Addressing the paucity of data on onychomycosis in South Africa is essential, as the absence of local epidemiological and mycological evidence necessitates empirical treatment that may be ineffective and contribute to poor outcomes. We therefore investigated the clinical spectrum, risk factors and mycological profile associated with onychomycosis in KwaZulu-Natal (KZN).

## Research methods and design

**FIGURE 1:**
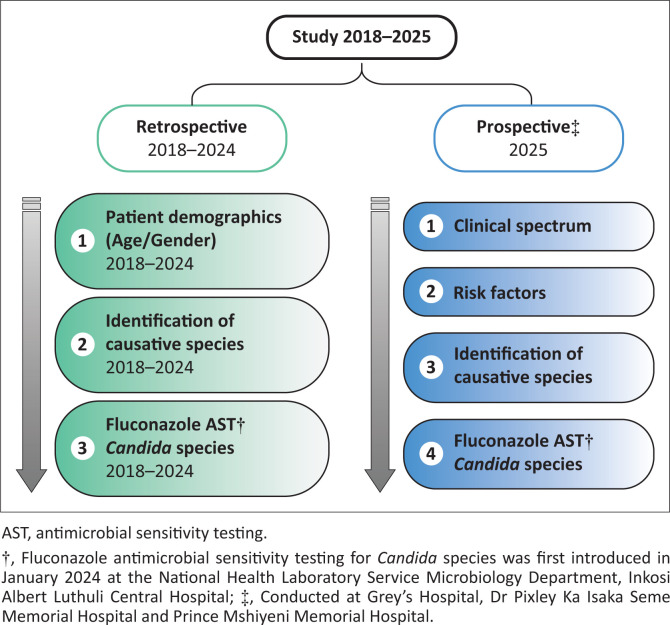
Study flow diagram.

The study was a quantitative, observational, descriptive study with both a retrospective and a prospective component, as illustrated in Figure 1.

### Retrospective component of the study

All positive cultures isolated from patients throughout KZN were analysed at the reference mycology laboratory of the National Health Laboratory Service (NHLS) in KZN from 2018 to 2024. The data obtained included patients’ demographics, causative species identified and fluconazole susceptibility data for *Candida* species for 2024.

### Prospective component of the study

Patients attending the dermatology clinic at three hospitals in KZN; Grey’s Hospital in Pietermaritzburg, Dr Pixley Ka Isaka Seme Memorial Hospital and Prince Mshiyeni Memorial Hospital in Durban, South Africa from January 2025 to September 2025, were screened for onychomycosis of toenails and fingernails. Inclusion criteria were adult patients (18 years old and older) attending dermatology clinics at the respective study sites, patients who consented to participate and patients with dystrophic nails clinically resembling onychomycosis.

After a thorough examination of all fingernails, toenails and surrounding skin, the clinical spectrum and extent of nail involvement were documented and the relevant nail specimens were taken. A large nail cutter (nail pliers or nipper for thick toenails), a scalpel blade for scraping the nail plate and a spoon excavator were used for the collection of the affected nail as well as subungual debris. The pieces of nail and debris were collected on a dry, clean paper and placed in a sterile container. The nail specimen was then sent to the microbiology laboratory, where it was processed according to standard operating protocols.

Microscopy was performed using potassium hydroxide, and samples were cultured on Sabouraud plus chloramphenicol slopes and Sabouraud slopes containing both chloramphenicol and actidione. For dermatophytes and moulds, cultures were identified by macroscopic and microscopic morphological characteristics. For yeasts, Vitek 2^®^ system (BioMérieux) was used for identification and fluconazole susceptibility testing. Susceptibility testing was only conducted from 2024.

The site, extent, clinical type of onychomycosis, prior antifungal therapy, microscopy, fungal identification and *Candida* species susceptibility results were documented. The risk factors associated with onychomycosis include HIV infection, diabetes mellitus, tinea pedis, psoriasis, hyperhidrosis, chronic venous insufficiency, peripheral artery disease, obesity, trauma to nails, wearing occlusive footwear, advanced age, contact with household members who suffer from onychomycosis or tinea pedis, hallux valgus, smoking, humidity, genetic predisposition and immunosuppression were documented for each patient. Immunosuppression was defined in this study as a decreased immunity secondary to chronic kidney disease, metabolic syndrome, malignancy and patients on an immunosuppressant. As standard practice, all patients with more than three nails involved were investigated for HIV infection, which included voluntary HIV counselling and testing (VCT test). CD4 count and viral load of HIV-positive patients were also recorded.

### Statistical analysis

The statistical data analysis was conducted in R Statistical^®^ computing software of the R Core Team, 2020, version 3.6.3 (R Foundation for Statistical Computing, Vienna, Austria) and visually displayed in Microsoft Excel 2021. The results were presented in the form of descriptive and inferential statistics. There were no numerical variables. The categorical variables were described as counts and percentage frequencies, where simple and multiple bar charts were used to visually display the categorical variables. To determine the association between categorical variables, a chi-square test was used, and when the distribution of the cross-tabulations contained an expected value of less than five, a Fisher’s exact test was applied. In the case of a significant difference between the chi-square or Fisher’s exact test, a row-wise paired *z*-test was used as a post hoc analysis following the omnibus tests (chi-square or Fisher’s exact test). All the inferential statistical analysis tests were conducted at 5% levels of significance.

### Ethical considerations

The study was conducted in accordance with stringent ethical standards, with all required procedures fully adhered to. Ethical approval was obtained from the University of KwaZulu-Natal Biomedical Research Ethics Committee (BREC/00008103/2024), the South African Department of Health National Health Research Ethics Council and NHLS Academic Affairs and Research Management system. Hospital gatekeeper approvals from Grey’s Hospital in Pietermaritzburg and Dr Pixley Ka Isaka Seme Memorial Hospital and Prince Mshiyeni Memorial Hospital in Durban were also obtained. Written consent was obtained from each participant, and all data were anonymised.

## Results

### Patients’ demographics

#### Retrospective study

**FIGURE 2:**
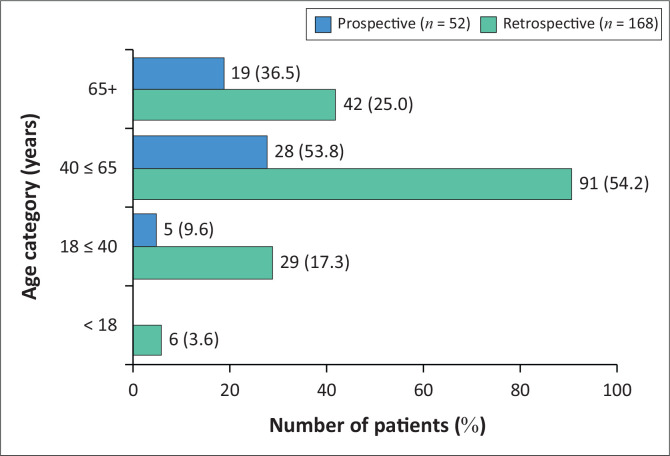
Age distribution of patients with positive microbiology culture: Retrospective study and prospective study.

Out of a total of 177 patients who were analysed, the age of 9 patients was unknown. The median age was 55.0 (43.0–64.3) years. The majority of patients belonged to the 40 years – < 65 years old (54.2%) age category compared to other age groups, and a higher distribution was noted in females (65.5%) compared to males (28.8%) (Figure 2).

#### Prospective study

A total of 124 patients were included with a median age of 58.0 (48.0–68.0) years. In the culture-positive group, a higher proportion of patients were between 40 years and < 65 years of age (53.8%) compared to other age groups (Figure 2).

Overall, 64 patients (51.6%) were female, and 60 patients (48.4%) were male. Of the 52 patients with positive microbiology culture, 29 patients (55.8%) were female, and 23 patients (44.2%) were male. There was no association between age and gender with regard to culture status.

### Spectrum of causative species identified

Of the 301 patients, 229 patients with positive cultures were included in the analysis: 177 patients from the retrospective study and 52 patients from the prospective study.

#### Retrospective study

**FIGURE 3:**
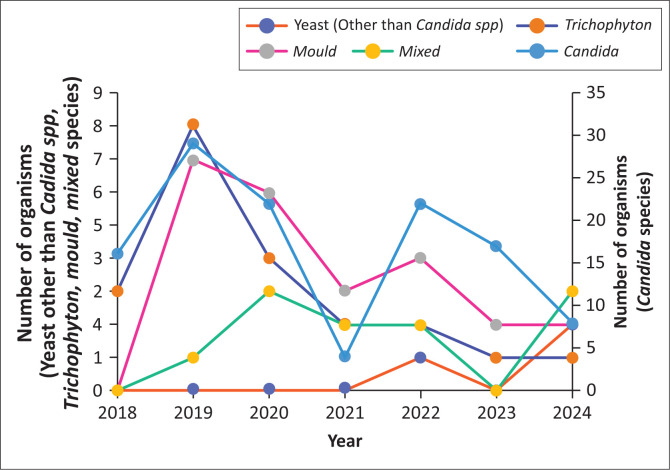
Retrospective study: Trend in the prevalence of organisms over the *years*.

The identified organisms were categorised as *Candida* species, moulds, *Trichophyton* species, yeast (other than *Candida* species) and mixed, as described in Table 1. *Candida* species were most commonly isolated (66.7%). Moulds and *Trichophyton* species accounted for 13.6% and 11.9%, respectively. Other dermatophytes were not isolated. The pattern of distribution of the species is demonstrated in Figure 3. A drop in patients with positive cultures was reported in 2020 and 2021, which coincides with the coronavirus disease 2019 (COVID-19) pandemic (Figure 3).

**TABLE 1:** Breakdown of positive microbiology culture for retrospective and prospective arms of the study.

Microbiology culture	Retrospective study Overall (*n* = 177)		Prospective study Overall (*n* = 52)	
	*n*	%	*n*	%
***Candida spp***	**118**	**66.7**	**21**	**40.4**
*Candida parapsilosis*	54	30.5	14	26.9
*Candida tropicalis*	10	5.6	-	-
*Candida albicans*	47	26.6	1	1.9
*Candida sake*	1	0.6	-	-
*Candida guilliermondii*	4	2.3	-	-
*Candida glabrata*	1	0.6	-	-
*Other Candida* species	1	0.6	6	11.5
***Trichophyton spp***	**21**	**11.9**	**13**	**25.0**
*Trichophyton rubrum*	4	2.3	5	9.6
*Trichophyton mentagrophytes*	5	2.8	2	3.8
*Trichophyton verrucosum*	1	0.6	-	-
*Trichophyton violaceum*	10	5.6	3	5.8
*Trichophyton tonsurans*	1	0.6	3	5.8
***Moulds***	**24**	**13.6**	**13**	**25.0**
*Scopulariopsis brevicaulis*	4	2.3	-	-
*Fusarium* species	6	3.4	2	3.8
*Geotrichum capitatum*	1	0.6	-	-
*Geotrichum* species	3	1.7	1	1.9
*Acremonium* species	1	0.6	1	1.9
*Aspergillus fumigatus*	1	0.6	-	-
*Aspergillus terreus*	1	0.6	-	-
*Aspergillus versicolor*	3	1.7	2	3.8
*Gliocladium* species	1	0.6	-	-
*Chrysosporium* species	1	0.6	3	5.8
*Scopulariopsis* species	1	0.6	3	5.8
*Paecilomyces* species	1	0.6	-	-
*Mould* species *unable to identify*	-	-	1	1.9
***Other yeast* species**	**3**	**1.7**	**-**	**-**
*Trichosporon asahii*	1	0.6	-	-
*Kodamaea ohmeri*	1	0.6	-	-
*Trichosporon* species	1	0.6	-	-
***Mixed***	**11**	**6.2**	**5**	**9.6**
*Candida parapsilosis; Fusarium* species	2	1.1	1	1.9
*Candida albicans; Candida parapsilosis*	3	1.7	-	-
*Acremonium* species; *Candida parapsilosis*	1	0.6	-	-
Candida parapsilosis; *Trichophyton violaceum*	2	1.1	-	-
*Candida parapsilosis; Trichophyton* species	1	0.6	-	-
*Candida albicans; Fusarium* species	1	0.6	-	-
*Aspergillus versicolor; Fusarium* species	1	0.6	-	-
*Fusarium* species; *Candida tropicalis*	-	-	1	1.9
*Trichophyton rubrum; Acremonium* species	-	-	1	1.9
*Candida parapsilosis; Trichophyton rubrum*	-	-	1	1.9
*Trichophyton tonsurans; Aspergillus versicolor*	-	-	1	1.9

#### Prospective study

Of the 124 patients, 52 patients (41.9%) had positive fungal cultures, and 72 (58.1%) were negative. Six patients (8.3%), out of the 72 patients who had negative fungal cultures, received prior antifungal therapy.

Out of the 52 patients who had positive fungal cultures, *Candida* species represented the largest proportion followed by *Trichophyton* species and moulds.

The distribution of all species for both arms of the study is illustrated in Table 1.

### Fluconazole antimicrobial sensitivity testing for *Candida* species

All *Candida* isolates tested were susceptible to fluconazole: 8 from the retrospective study and 17 from the prospective study.

### Direct microscopy

In the prospective study, 101/124 patients (81.5%) had negative microscopy results, as summarised in Table 2. Similarly, even among the 52 positive culture patients, the majority had negative microscopy results: 42/52 (80.8%).

**TABLE 2:** Prospective study: Microscopy and microbiology status.

Microscopy	Microbiology culture status						*p*-value*
	Negative (*n* = 72)		Positive (*n* = 52)		Overall (*n* = 124)		
	*n*	%	*n*	%	*n*	%	
Negative	59	81.9	42	80.8	101	81.5	0.334
Fungal hyphae	11	15.3	8	15.4	19	15.3	-
Fungal hyphae; Yeast	2	2.8	-	-	2	1.6	-
Yeast	0	0.0	2	3.8	1	1.6	-

* , Fischer’s exact test.

Although 72 patients had negative microbiology cultures, 13 of these had a positive microscopy, and 2 patients out of these 13 cases (15.4%) received prior antifungal therapy.

In the culture-positive group, 10 patients out of the 42 patients with negative microscopy (23.8%) had received prior antifungal therapy. The majority of the patients with positive microscopy were observed to have fungal hyphae (15.3% overall). No significant association was observed between microscopy findings and positive fungal cultures.

### Clinical spectrum

**FIGURE 4:**
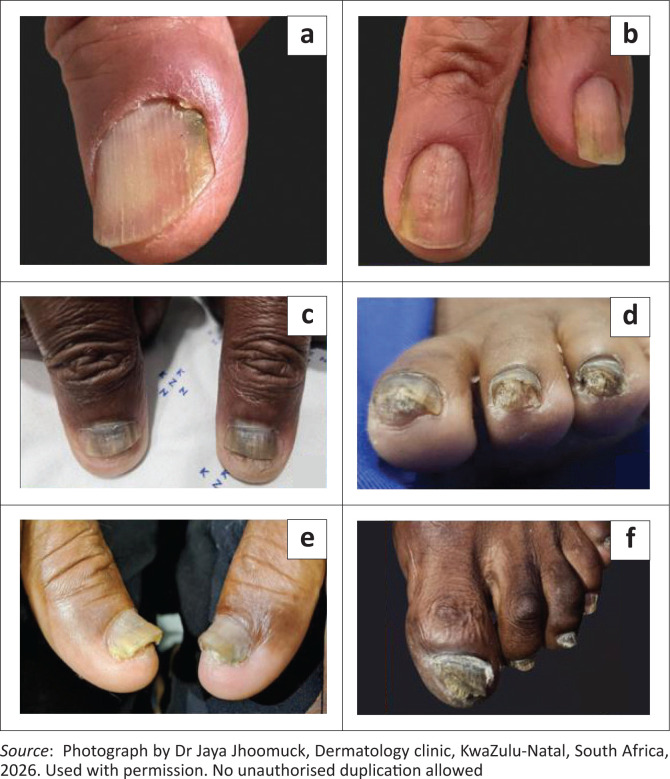
*(a-f) Types of onychomycosis and nail discolouration in our study population. (a, b) Distal lateral onychomycosis. (a, b) Paronychia. (c, d, e, f) Total nail dystrophy. (d, e) Subungual hyperkeratosis. (c, f) Black discolouration. (d) Brown discolouration. (e) Yellow discolouration*.

In our setting, we found a higher proportion of patients presented with subungual hyperkeratosis (69.2%) in the positive microbiology culture arm compared to other types of onychomycosis as outlined in Table 3 and illustrated in Figure 4. Most patients presented with black discolouration of the nails.

**TABLE 3:** Prospective study: Clinical findings.

Clinical findings	Microbiology negative culture (*n* = 72)		Microbiology positive culture (*n* = 52)		Overall (*n* = 124)		*p*-value*
	*n*	%	*n*	%	*n*	%	
**Type of onychomycosis**	-	-	-	-	-	-	0.476
Subungual hyperkeratosis	51	70.8	36	69.2	87	70.2	-
Distal subungual	3	4.2	4	7.7	7	5.6	-
Proximal subungual	1	1.4	0	0.0	1	0.8	-
White superficial	3	4.2	0	0.0	3	2.4	-
Subungual hyperkeratosis; White superficial	0	0.0	1	1.9	1	0.8	-
Distal lateral	14	19.4	11	21.2	25	20.2	-

* , Fischer’s exact test.

### Risk factors

#### Human immunodeficiency virus and onychomycosis

Thirty-one patients (25.0%) were HIV-positive, while 93 patients (75.0%) were HIV-negative in the prospective study. Of the HIV-infected patients, 14 patients (45.2%) had a positive microbiology culture, and 17 patients (54.8%) had a negative microbiology culture. The breakdown of CD4 count and viral load is categorised as illustrated in Table 4. We observed that the prevalence of positive cultures was higher in virologically suppressed HIV-infected patients (CD4 count between 500 cells/µL and 750 cells/µL and those with undetectable viral loads). There was no difference noted in the culture positivity between HIV-positive and HIV-negative patients.

**TABLE 4:** Prospective study: CD4 count and viral load of human immunodeficiency virus-positive patients with positive and negative cultures.

CD4 count and Viral load	Microbiology positive culture (*n* = 14)		Microbiology negative culture (*n* = 17)		Overall (*n* = 31)	
	*n*	%	*n*	%	*n*	%
**CD4 count (cells/µL)**						
< 250	-	-	3	17.6	3	9.7
250 – < 500	1	7.1	4	23.5	5	16.1
500 – < 750	10	71.4	4	23.5	14	45.2
750–1000	1	7.1	3	17.6	4	12.9
> 1000	2	14.3	3	17.6	5	16.1
**Viral load (copies/mL)**						
Lower than detectable	9	64.3	6	35.3	15	48.4
< 250	4	28.6	7	41.2	11	35.5
250 – < 500	-	-	1	5.9	1	3.2
> 1000	1	7.1	3	17.6	4	12.9

Eight HIV-infected patients with positive microbiology culture (57.1%) presented with subungual hyperkeratosis, followed by five patients (35.7%) with distal lateral onychomycosis and one patient (7.1%) with distal onychomycosis. The prevalence of organisms identified within the HIV-infected patients is illustrated in Online Appendix 1, Table 1-OA1.

#### Various risk factors and fungal aetiology

Among the patients with a positive microbiology culture, a higher prevalence of immunosuppression (40.4%) was observed, followed by other risk factors such as HIV infection (26.9%), tinea pedis (25.0%), diabetes mellitus (17.3%), psoriasis (13.5%) and trauma to nails (5.8%). No association was noted between microbiological culture categories and risk factors (Online Appendix 1, Table 1-OA1).

Other risk factors were investigated, which included hyperhidrosis, chronic venous insufficiency, peripheral artery disease, obesity, wearing occlusive footwear, advanced age, contact with household members who suffer from onychomycosis or tinea pedis, hallux valgus, smoking, humidity and genetic predisposition, and no significant association was observed (Online Appendix 1, Table 2-OA1).

#### Other comorbidities association with microbiology culture status

In the culture-positive group, 44 patients had additional dermatological or occupational comorbidities documented during clinical assessment. Stasis eczema was the most frequently observed comorbidity, present in 15 patients (34.1%) (Online Appendix 1, Figure 1-OA1).

## Discussion

We studied patients’ demographics, risk factors, clinical spectrum and microbiological aspects of onychomycosis in KZN. Our study found that most patients presented with subungual hyperkeratinisation and immunosuppression (other than HIV infection and diabetes mellitus) was the most common risk factor. *Candida* species were the predominant cause of onychomycosis, and all *Candida* isolates tested were susceptible to fluconazole, an antifungal usually prescribed in our setting.

Current literature reports a 20% – 50% higher prevalence of onychomycosis in older adults than in other age groups.^14^ Older adult patients tend to have poor foot hygiene resulting from many comorbidities (such as eye diseases, arthritis of the hands) and mobility problems.^14^ In contrast, our study reported a higher prevalence in the age category between 40 and < 65 years old compared to the age category of more than 65 years old. This may reflect differences in the health-seeking behaviour of patients attending our clinics, with poorer attendance by older patients.

Onychomycosis was found to be more common in females compared to males in our retrospective study, and a negligible difference was noted in the prospective study. Global male-to-female prevalence ratios of onychomycosis range from 0.84 to 1.72, and the lower prevalence in females is not clear, which warrants further investigation.^15,16^ The predominance in female gender in the retrospective arm could reflect a greater health-seeking behaviour and greater concern about cosmetic appearance compared to men. In addition, because patients were actively being screened for onychomycosis in the prospective study, the male prevalence showed an increase and was comparable to that of females, suggesting that male patients may be less likely to seek treatment for their nail condition. Current literature reports men being less likely to seek help for other health issues, such as mental health issues.^17^

A high percentage of microscopy-negative specimens (80.8%) had a positive microbiological culture in our prospective study, with only 14.5% receiving prior antifungal therapy that could have influenced the result. Although microscopy is quick and inexpensive, it is known to have a variable sensitivity of 33.7% – 93% and specificity from 38% to 100%.^18^ Our study confirms this variability and therefore, microscopy should not be used in isolation to exclude onychomycosis.

Furthermore, our prospective study demonstrated that 41.9% patients had positive cultures, which is in keeping with the 43.2% in another cross-sectional study.^19^ Possible attributable reasons for this higher yield compared to our previous pick-up rate of 16.7% (unpublished data) in our setting could be because of a thorough examination to select the nail sampling site and sampling from a deeper proportion of affected nails to obtain a representative specimen. A total of 58.1% patients with negative cultures could be related to false-negative cultures. This diagnostic gap is largely attributed to improper specimen collection, where the sample may contain insufficient fungal load, non-viable organisms or contaminants,^20^ and other causes could also have been secondary to prior antifungal use and loss of viability in transit. A Canadian study reported that culture was three or four times more likely to report a false-negative result compared with Polymerase Chain Reaction (PCR) and also recommended serial samples to help confirm the diagnosis of NDM infections.^21^ It is also indicative that patients may have a non-infective aetiology. This highlights the indispensable value of microbiological culture testing in confirming the diagnosis and also the importance of enlarging our clinical differentials when encountering a clinical case of onychomycosis. In another study, only half of the nail specimens submitted to a US diagnostic laboratory tested positive for fungal infection despite a clinical diagnosis of onychomycosis, further highlighting the need to confirm the diagnosis under the tenets of antifungal stewardship.^22^ Interestingly, there was a reduced number of patients with positive microbiology culture recorded in the retrospective study for the years 2020 and 2021. This may be attributed to reduced access and attendance to hospitals and outpatient clinics during the COVID-19 pandemic.

The current body of literature reports considerable variation in the prevalence of identified organisms. We found a predominance of *Candida* species (particularly *C. parapsilosis*), followed by an almost equal distribution of mould and *Trichophyton* species. Our results are in keeping with a 12-year study carried out in Greece, where yeasts, followed by dermatophyte species and NDMs were most frequently diagnosed.^23^ Brilhante et al. also reported that yeast infection was the most commonly detected infection, especially in fingernail onychomycosis.^24^ However, Gupta et al. reported dermatophytes and yeasts at equal rates, while Mügge et al. reported dermatophytes as the most common pathogen.^25,26^ Recently, NDMs accounted for approximately 6.9% of onychomycosis cases globally, 3.3% in North America and between 10.1% and 23.5% in Central Asia and West Asia.^27^ Gupta et al. also postulated that the lower detection rate of mould in Africa (4.6%) is likely an artefact of significant variations in reported rates across studies.^27^ Our study supports this theory as mould prevalence was found to be higher: 13.6% in our retrospective study and 25.0% in our prospective study. The higher rates of detection may partially be attributed to a relatively warm, humid climate favouring the growth of NDMs, which may manifest as mixed pattern onychomycosis.^27^ Another study from Ireland highlighted that out of a total of 8645 patients with onychomycosis, the majority were found to have *T. rubrum* isolated followed by yeast as the second most common organism.^16^ Rare species such as *Epidermophyton flocossum* was also identified in 15 patients in the same study, which is in contrast to our study where *Candida* species predominated and *Epidermophyton* species were not isolated.^16^

Fluconazole resistance was not detected in all *Candida* isolates tested in our study. Conversely, another study reported 12.9% *C. albicans* were resistant to fluconazole.^28^ In addition, that study demonstrated fluconazole resistance in 31.3% and 25.9% *C. parapsilosis* and *Candida tropicalis*, respectively.^28^ The emergence and global spread of antifungal resistance among species of *Trichophyton* and resistant yeast and mould species associated with systemic infection are now categorised by the World Health Organization as fungal pathogens that represent a great threat to public health.^29^ In this era of global antifungal resistance,^29^ our data show that fluconazole susceptibility to *Candida* species was still preserved locally. As fluconazole is the only antifungal agent available to treat onychomycosis in our setting, it is used to treat all cases of positive microbiology culture cases. While the findings of our study support the use of fluconazole for onychomycosis with *Candida* species, it is not active against *Trichophyton* species and moulds, and this may explain the numerous difficult-to-treat onychomycosis cases encountered in our clinics.

Subungual hyperkeratosis was the most common type of onychomycosis (70.2%) and was higher in prevalence among the microbiology-positive cultures, followed by distal lateral and distal subungual onychomycosis. Saxena et al. reported distal lateral subungual onychomycosis as the most prevalent in patients from the dermatology outpatient department in India, followed by total dystrophic onychomycosis and PSO.^5^ This demonstrated a variation in the pattern of distribution in our setting in KwaZulu-Natal, South Africa. The plausible factor contributing to this finding could be that onychomycosis is often overlooked, and patients seek medical treatment only once the condition progresses to full-thickness subungual hyperkeratosis.

KwaZulu-Natal has the second-highest HIV prevalence rate in South Africa in 2024^30^; therefore, our study provides valuable information on the potential association of HIV with onychomycosis. Proximal subungual onychomycosis is associated with immunosuppression and regarded as a sign of HIV infection.^31^ Our findings differed as the majority of HIV-infected patients with positive microbiology presented with subungual hyperkeratosis and none with PSO. Although Aggarwal et al. report a 25% higher prevalence of onychomycosis in HIV-positive patients,^1^ this KZN-based study shows, contrary to expectations, no difference in prevalence between the HIV-positive and HIV-negative patients in relation to positive microbial culture. Surprisingly, there was a higher prevalence of positive culture observed in virologically suppressed HIV-infected patients (CD4 count between 500 cells/µL and 750 cells/µL and a viral load of lower than detectable categories), which would reflect an effective antiretroviral coverage. A possible reason could be immune reconstitution inflammatory syndrome (IRIS), an inflammatory response because of immune rebound, which can occur in people treated with antiretrovirals, particularly those with active opportunistic infections.^32^ Current literature reports two suspected tinea-IRIS cases from an HIV clinic in Tanzania;^33^ it is possible that similar mechanism may underlie some cases of onychomycosis. Also, fluconazole should be used with caution in HIV-positive patients because of potential drug–drug interactions.^34^

Immunosuppression (secondary to causes other than HIV infection and diabetes mellitus) was the most common risk factor, and trauma to nails was the least common. Although there are several other risks, our prospective study did not show any. We postulate that these risk factors are not independent predictors of onychomycosis but rather belong to a more complex, multifactorial and ecologically driven disease instead of being a linear one.

Multiple comorbidities that could potentially be associated with onychomycosis were also highlighted in this study. Co-existing conditions, such as stasis eczema, tinea corporis and varicose veins, as well as occupational history, were found to be more common. The literature mentions that abnormal nail changes such as brittleness associated with chronic venous disease can also increase infection risk, and an upregulated pro-inflammatory immune response may offset the risk of infection.^35^ This suggests that the pathogenesis of these conditions may directly or indirectly be associated with increased risk of developing onychomycosis.

### Recommendations

This study recommends:

- Thorough examination, sampling from a deeper proportion of affected nails to obtain a representative specimen and repeated nail culture are recommended to rule out false-negative cultures.
- Revision of the existing essential drug list is warranted to include the use of itraconazole or terbinafine for onychomycosis patients with confirmed mould species on culture.
- Continue using fluconazole in confirmed *Candida* species cases.
- Ongoing surveillance of susceptibility patterns, including dermatophytes and moulds, is recommended.
- Co-existing conditions (such as stasis eczema, tinea corporis and varicose veins and occupational history) were found to be more common. Future research could investigate other possible underlying pathways of onychomycosis, such as inflammation or barrier dysfunction that could act as a contributory factor.

### Limitations

Culture-based diagnostics were used in the study rather than molecular assays, and this may account for the high number of culture-negative cases. Fluconazole AST for *Candida* species was not conducted prior to 2024; therefore, fluconazole AST data were unavailable for earlier years. Susceptibility testing of dermatophytes and moulds was also not conducted routinely. The study was potentially influenced by the attendance pattern of patients attending the clinic.

## Conclusion

To the best of our knowledge, our study provides the first detailed analysis of onychomycosis in KZN, South Africa, building a baseline for an almost non-existent database on onychomycosis. By defining the local microbiological profile and highlighting key gaps in diagnosis and management, we offer actionable recommendations and a clear agenda for future research; notably, phenotypes of onychomycosis in HIV-infected patients, the spectrum of clinical mimickers of onychomycosis, serial sampling influence on diagnostic yield, the role of other comorbidities in the pathogenesis of onychomycosis and a South African algorithm to treat onychomycosis. Strengthening and expanding local surveillance is pivotal to shift practice from empirical prescribing to culture- and susceptibility-guided, evidence-based treatment – improving outcomes, reducing recalcitrant disease and supporting responsible antifungal use.
